# Ecto- and arbuscular mycorrhizal symbiosis can induce tolerance to toxic pulses of phosphorus in jarrah (*Eucalyptus marginata*) seedlings

**DOI:** 10.1007/s00572-014-0567-6

**Published:** 2014-03-02

**Authors:** Khalil Kariman, Susan J. Barker, Patrick M. Finnegan, Mark Tibbett

**Affiliations:** 1 School of Earth and Environment M087, The University of Western Australia, Crawley, WA 6009 Australia; 2 School of Plant Biology M084, The University of Western Australia, Crawley, WA 6009 Australia; 3 Institute of Agriculture M082, The University of Western Australia, Crawley, WA 6009 Australia; 4 Department of Environmental Science and Technology (B37), School of Applied Sciences, Cranfield University, Cranfield, Bedfordshire, MK 43 OAL England

**Keywords:** Arbuscular mycorrhiza (AM), Ectomycorrhiza (ECM), Jarrah, Phosphorus (P) toxicity, Tolerance, P pulse

## Abstract

In common with many plants native to low P soils, jarrah (*Eucalyptus marginata*) develops toxicity symptoms upon exposure to elevated phosphorus (P). Jarrah plants can establish arbuscular mycorrhizal (AM) and ectomycorrhizal (ECM) associations, along with a non-colonizing symbiosis described recently. AM colonization is known to influence the pattern of expression of genes required for P uptake of host plants and our aim was to investigate this phenomenon in relation to P sensitivity. Therefore, we examined the effect on hosts of the presence of AM and ECM fungi in combination with toxic pulses of P and assessed possible correlations between the induced tolerance and the shoot P concentration. The P transport dynamics of AM (*Rhizophagus irregularis* and *Scutellospora calospora*), ECM (*Scleroderma* sp.), non-colonizing symbiosis (*Austroboletus occidentalis*), dual mycorrhizal (*R. irregularis* and *Scleroderma* sp.), and non-mycorrhizal (NM) seedlings were monitored following two pulses of P. The ECM and *A. occidentalis* associations significantly enhanced the shoot P content of jarrah plants growing under P-deficient conditions. In addition, *S. calospora*, *A. occidentalis*, and *Scleroderma* sp. all stimulated plant growth significantly. All inoculated plants had significantly lower phytotoxicity symptoms compared to NM controls 7 days after addition of an elevated P dose (30 mg P kg^−1^ soil). Following exposure to toxicity-inducing levels of P, the shoot P concentration was significantly lower in *R. irregularis*-inoculated and dually inoculated plants compared to NM controls. Although all inoculated plants had reduced toxicity symptoms and there was a positive linear relationship between rank and shoot P concentration, the protective effect was not necessarily explained by the type of fungal association or the extent of mycorrhizal colonization.

## Introduction

Phosphorus (P) is a macronutrient essential for plant growth. It is a structural component of nucleic acids and phospholipids, and is involved in many cellular functions such as energy transfer and the regulation of enzyme activity. The availability of P to plants is, however, limited in many soils. Australia, sub-Saharan Africa, tropical Asia and South America are among the main areas that have P-deficient soils (Sanchez and Buol 1975; Runge-Metzger 1995; Handreck 1997; Trolove et al. 2003). P deficiency is considered to be the main factor determining plant productivity and species diversity in ancient landscapes (Lambers et al. 2010).

Many plant species have mechanisms to enhance the extraction of P from the soil. Arbuscular mycorrhiza (AM), ectomycorrhiza (ECM) and cluster root formation are among the main P acquisition strategies available to plants (Lambers and Shane 2007; Smith and Read 2008; Lambers et al. 2009). AM fungi are found in the majority of terrestrial ecosystems. In a survey of the literature, they were shown to colonize 74 % of angiosperm species from 336 plant families representing 99 % of flowering plants (Brundrett 2009). ECM fungi establish the second most widespread form of mycorrhiza and have an intimate association with many woody plant species from about 30 families (Smith and Read 2008).

Plant species that are adapted to P-deficient soils can be exposed to elevated P conditions via nutrient flushing, soil wetting, fertilization and also when new plantations are established on previously fertilized soils having high levels of P (Handreck 1997; Trolove et al. 2003). Plants that naturally grow in low P soils, particularly those from Australia and South Africa, can develop toxicity symptoms upon exposure to elevated levels of P (Shane et al. 2004a, b; Hawkins et al. 2008). The P toxicity response occurs in at least some low-P adapted species because they cannot down-regulate their net P uptake, perhaps as a consequence of having evolved on P-impoverished soils over millions of years (Shane et al. 2004a; Hawkins et al. 2008; Lambers et al. 2011). P-sensitive species are found in the Fabaceae, Haemodoraceae, Mimosaceae, Myrtaceae, Proteaceae, Rutaceae, and generally are a feature of heaths and other sclerophyllous plant communities (Specht and Groves 1966; Grundon 1972; Heddle and Specht 1975; Specht et al. 1977; Specht 1981; Dell et al. 1987; Handreck 1997; Shane et al. 2004b; Thomson and Leishman 2004; Hawkins et al. 2008). Depending on the plant species (Shane et al. 2004b), development of P toxicity symptoms can occur at a shoot P concentration of less than 1 mg P g^−1^ DW, such as in *Banksia ericifolia* (Parks et al. 2000), or more than 40 mg P g^−1^ DW, as reported for *Telopia speciosissima* (Grose 1989).

The response of plants to P fertilization may be linked to their response to root colonization by mycorrhizal fungi, but the details of this relationship are not clear. Although mycorrhizal symbioses are renowned for increasing nutrient uptake in nutrient-deficient plants, they also function to favour the growth of plants exposed to toxic concentrations of heavy metals or certain essential trace elements such as Zn (Jentschke and Godbold 2000; Hildebrandt et al. 2007). Mycorrhizal fungi can modify the P uptake in the host plant by inducing the plant to reduce the expression of genes encoding high-affinity phosphate transporter (PHT) proteins (Liu et al. 1998; Burleigh and Harrison 1999; Rosewarne et al. 1999; Burleigh 2001; Karandashov and Bucher 2005). Much more information about shoot P accumulation and toxicity development is required for plants that are commonly used in combination with P fertilizer in the restoration of native ecosystems (Koch 2007).

This research was carried out to clarify the possible link between mycorrhizal associations and P tolerance in *Eucalyptus marginata* (jarrah), an important species in forest restoration, suspected of high P sensitivity. The present study is the second stage of an experiment of which the first stage has been published elsewhere (Kariman et al. 2012). A nurse-pot system was used to establish mycorrhizal associations of jarrah with the AM species *R. irregularis* (Błaszk., Wubet, Renker & Buscot) C. Walker & A. Schüßler comb. nov. and *S. calospora* Nicol. & Gerd., the ECM fungus *Scleroderma* sp., a dual (AM and ECM) treatment of *R. irregularis* and *Scleroderma* sp. and a non-colonizing fungus *A. occidentalis* Watling & N.M. Greg. As previously described (Kariman et al. 2012), the mycorrhizal colonization of nurse seedlings were 2.3, 29 and 28.3 % for *R. irregularis* (AM), *S. calospora* (AM) and *Scleroderma* sp. (ECM) treatments, respectively. The dual treatment had less than 1 % AM and no ECM colonization, and *A. occidentalis* did not colonize jarrah roots. The positive growth responses were observed even when only one replicate was colonized (out of three, *Scleroderma* sp.) or there was no sign of root colonization (*A. occidentalis*). Our subsequent study unearthed a novel plant–fungus symbiosis between jarrah and *A. occidentalis*, in which plant growth and nutrient acquisition is substantially improved without forming mycorrhizal structures (Kariman et al. 2014). It is now more evident that root colonization is not necessarily required for positive physiological responses in plant–fungus associations (Neumann 1959; Kariman et al. 2012, 2014). In the current study, we explored AM, ECM and the *A. occidentalis* associations in jarrah seedlings along the P toxicity continuum. The main aims of this study were (i) to establish the role of the fungi in stimulating P uptake under P-deficient conditions; (ii) to determine the ability of the selected fungi to confer tolerance against toxic pulses of P; (iii) to reveal possible correlations between the induced tolerance and the shoot P concentration, the type of fungal association and the extent of root colonization and (iv) to ascertain the effect of these fungal species on plant growth.

## Materials and methods

### Plant materials, fungal isolates and inoculum production

Jarrah capsules were obtained from a single tree near Dwellingup, Western Australia. Seeds were released from the capsules by incubating at 42 °C for 3 days. The four fungal isolates used were *S. calospora*, *A. occidentalis* and *Scleroderma* sp. from west Australian habitats and *R. irregularis* (*DAOM197198*), an exotic isolate from Pont Rouge, Québec, Canada (Stockinger et al. 2009). Details about the fungal isolates and inoculum production were as previously described (Kariman et al. 2012).

### Nurse-pot system

A nurse-pot system was developed for the study of dual mycorrhizal associations of jarrah seedlings (Kariman et al. 2012). A polyester mesh bag (diameter and depth, 17 by 17 cm) with 40 μm pore size was filled with 3.75 kg of double-pasteurized washed river sand and placed in the centre of a plastic pot (diameter and depth, 23 by 25 cm). The matrix outside the mesh bag was filled with 4.5 kg of a mixture of double-pasteurized washed river sand and mycorrhizal inoculums. AM inocula (*R. irregularis* and *S. calospora*) were bulked by growing leek plants in a mixture of AM inoculum and double-pasteurized washed river sand (1:9 *w*/*w*) for 4 months. A vermiculite-based medium was used to produce hyphal inoculum for *Scleroderma* sp. and *A. occidentalis* (see Kariman et al. 2012 for more details). To provide equivalent conditions for all treatments, AM plants received sterilized ECM inoculum and ECM- and *A. occidentalis*-treated plants were supplied with sterilized AM inoculum. Non-mycorrhizal (NM) plants also received sterilized AM and ECM inocula. The dual treatment was supplied with *R. irregularis* (AM) and *Scleroderma* sp. (ECM) inocula. Three replicate nurse-pots were considered for each treatment in a completely randomized design. Four pre-germinated jarrah seeds were planted outside the mesh bag and designated as nurse seedlings. Our preliminary experiment revealed that jarrah plants would be colonized 10 weeks after inoculation with mycorrhizal fungi. Therefore, after 10 weeks growth of nurse seedlings, four NM test seedlings of the same age as nurse seedlings were transplanted into the mesh bag and one test seedling was harvested from each nurse-pot every 7 days to check the colonization, with the final test seedling removed at week 14. Table 1 provides the mycorrhizal colonization of the final test seedling and the first nurse seedling at week 14, both of which were harvested 1 day before addition of the first P pulse (Kariman et al. 2012). The present study deals with nurse seedlings after removing all four test seedlings from nurse-pots at the end of week 14. Hence, we use term “seedling” to refer to “nurse seedlings” in this manuscript. The AM colonization was measured using the gridline intersect method and at least 300 intersects were examined per sample (Giovannetti and Mosse 1980). The ECM colonization was quantified by determining the percentage of ECM root tips (Gehring and Whitham 1994) and a minimum of 500 root tips were counted per sample. The experiment was conducted from June to September 2010 in an unheated glasshouse with the average daytime temperature of 20 °C. All plants received the 1× modified Long Ashton solution minus P (10 mL kg^−1^ soil) once a fortnight started 2 weeks after planting: K_2_SO_4_ 2 mM, MgSO_4_ · 7H_2_O 1.5 mM, CaCl_2_ · 2H_2_O 3 mM, FeEDTA 0.1 mM, (NH_4_)_2_SO_4_ 4 mM, NaNO_3_ 8 mM, H_3_BO_3_ 46 μM, MnCl_2_ · 4H_2_O 9 μM, ZnSO_4_ · 7H_2_O 8 μM, CuSO_4_ · 5H_2_O 0.3 μM and Na_2_MoO_4_ · 2H_2_O 0.01 μM (Cavagnaro et al. 2001).

**Table 1:** Mycorrhizal colonization of 14-week-old test and nurse seedlings 1 day before addition of the first P pulse (10 mg P kg^−1^ soil), as also reported in Kariman et al. 2012

Treatments	Percentage of colonization	
	Test seedlings	Nurse seedlings
NM controls	NC	NC
*Rhizophagus irregularis* (AM)	NC	2.3 ± 0.8
*Scutellospora calospora* (AM)	8.2 ± 6.3	29 ± 4.8
*Austroboletus occidentalis*	NC	NC
*Scleroderma* sp. (ECM)	28.9 ± 28.9	28.3 ± 28.3
Dual: *Rhizophagus irregularis* (AM)	NC	0.8 ± 0.6
& *Scleroderma* sp. (ECM)	NC	NC

*NC*, no colonization, values are means ± SE (*n* = 3)

### P addition and toxicity analysis

One 14-week-old seedling from the former nurse treatments was harvested from each pot for shoot P analysis before addition of P pulses. The washed river sand used for plant culture contained less than 6 mg P kg^−1^ (data not shown). Therefore, the seedlings grown in the absence of P addition were grown under P-deficient conditions according to previous reports on eucalypts (Burgess et al. 1994; Aggangan et al. 1996). One day after harvesting the first seedling, the first P pulse was added to all pots at the ratio of 10 mg P kg^−1^ soil (as KH_2_PO_4_ in aqueous solution). One seedling was harvested from each pot 1 day after addition of the P pulse to measure the shoot P concentration. Seven days later, a second P pulse of 30 mg P kg^−1^ soil was added. The last seedling (16 weeks old) was harvested from all pots 1 week after adding the second P pulse to quantify P toxicity symptoms and investigate growth response and P accumulation under high P conditions. The P toxicity symptoms (including chlorotic and necrotic areas on leaves) were quantified by ranking plants into six classes from 0 to 5, where 0 corresponded to the absence of toxicity symptoms, 1 from traces to 20 % of symptomatic leaf tissue area (SLTA), 2 from 20 to 40 % SLTA, 3 from 40 to 60 % of SLTA, 4 from 60 to 80 % of SLTA and 5 more than 80 % of SLTA.

Measured quantities of ground dried shoot tissues (about 200 mg) were digested in 5 mL nitric–perchloric acid solution (4:1 *v*/*v*) and the P concentration was determined using a vanado-molybdate yellow method (Jackson 1973). The amount of P that accumulated in the shoot tissues (mg P g^−1^ DW) following each P pulse was calculated based on the differences in shoot P concentration between two subsequent harvests and was designated as the incremental shoot P concentration.

### Experimental design and data analysis

The experiment was conducted in a completely randomized design with three replicates. There were two AM treatments (*R. irregularis* and *S. calospora*), a non-colonizing treatment (*A. occidentalis*), an ECM treatment (*Scleroderma* sp.), a dual treatment (*R. irregularis* and *Scleroderma* sp.) and NM controls. One-way ANOVA and correlation analysis were performed using the Statistical Analysis System (SAS) version 9.2 (SAS Institute, Inc.; Cary NC, USA) software package. Means were separated using LSD at *p* < 0.05 in all datasets except for biomass data, where we used two *p* levels (*p* < 0.05 and *p* < 0.10).

## Results

### The ECM and non-colonizing fungi enhanced P uptake under P-deficient conditions

**Fig. 1:**
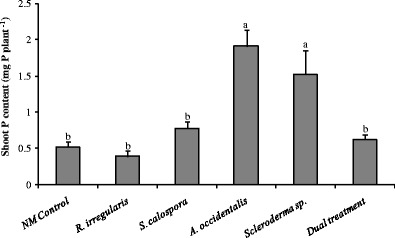
Shoot P content of jarrah seedlings growing under P-deficient conditions after 14 weeks growth (*p* = 0.0003). Plants were inoculated with the indicated fungi (except the un-inoculated control plants). The dual treatment was co-inoculated with *R. irregularis* (AM) and *Scleroderma* sp. (ECM). *Bars labelled with different letters* are significantly different at *p* < 0.05. *Error bars* are SE (*n* = 3)

We assessed the shoot P content of 14-week-old seedlings (former nurse seedlings) prior to the addition of P to study the effect of fungal treatments on P nutrition under P-deficient conditions (Fig. 1). Jarrah plants inoculated with the ECM fungus (*Scleroderma* sp.) and the non-colonizing fungus (*A. occidentalis*) had significantly higher shoot P content than the NM controls and the other inoculated treatments (*p* < 0.05).

### P sensitivity in jarrah is dependent on mycorrhizal fungi

**Fig. 2:**
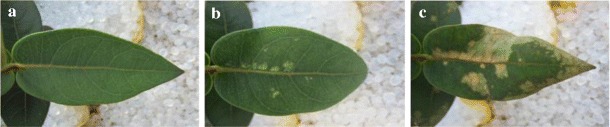
Development of P toxicity symptoms on an individual leaf from an NM jarrah seedling: **a** 1 day before (rank 0); **b** 3 days after (rank 1); and **c** 7 days after the second P dose (rank 3). Fourteen-week-old jarrah seedlings were exposed to a single dose of 10 mg P kg^−1^ soil for 7 days before subjecting to a second dose of 30 mg P kg^−1^ soil for 7 days

**Fig. 3:**
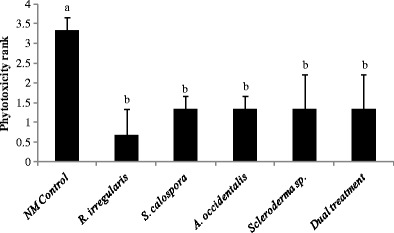
The average phytotoxicity rank (*p* = 0.1310) of 16-week-old jarrah plants 7 days after subjecting to the second dose of P (30 mg P kg^−1^ soil). Fourteen-week-old jarrah seedlings were exposed to a single dose of 10 mg P kg^−1^ soil for 7 days before subjecting to the second dose of P. The P toxicity symptoms were quantified by ranking plants into six classes from 0 to 5, where 0 corresponded to the absence of toxicity symptoms, 1 from traces to 20 % of symptomatic leaf tissue area (SLTA), 2 from 20 to 40 % SLTA, 3 from 40 to 60 % of SLTA, 4 from 60 to 80 % of SLTA and 5 more than 80 % of SLTA. Plants were inoculated with the indicated fungi and grown as described in the legend for Fig. 1. *Bars labelled with the same letter* are not significantly different at *p* < 0.05. *Error bars* are SE (*n* = 3)

Fourteen-week-old jarrah seedlings developed mild and patchy symptoms of P toxicity within 3 days of being exposed to 10 mg P kg^−1^ soil. The symptoms were more severe in plants exposed to a higher dose of 30 mg P kg^−1^ soil 7 days after the first dose. Irregular chlorotic spots appeared mainly around the midrib and progressed toward the leaf margins (Fig. 2). However, the pattern and development of symptoms differed among individual plants. All 16-week-old plants inoculated with live fungi had significantly reduced toxicity symptoms (*p* < 0.05) when examined 7 days after the second P addition compared to NM control plants (Fig. 3). The extent of the toxicity symptoms did not differ between plants inoculated with different fungi.

### Mycorrhizal fungi ameliorate the toxic accumulation of P in jarrah shoots

**Fig. 4:**
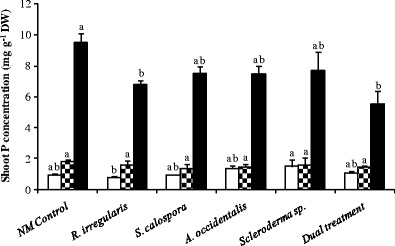
Shoot P concentration of jarrah seedlings 1 day before first P addition (*open bars*, *p* = 0.1434), 1 day after first P addition (*checkered bars*, *p* = 0.8798) and 7 days after the second P addition (*closed bars*, *p* = 0.0400). Plants were inoculated with fungi and grown as indicated in the legend to Fig. 1. *Bars from each harvest labelled with different letters* are significantly different at the *p* < 0.05 level. *Error bars* are SE (*n* = 3)

**Fig. 5:**
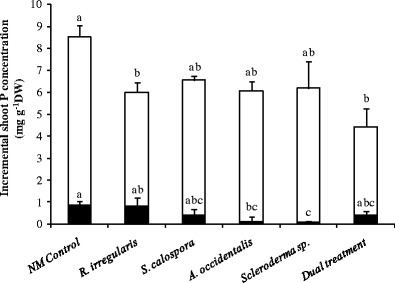
Incremental increase in shoot P concentration for jarrah seedlings 1 day after the first P addition (*closed bars*, *p* = 0.1391) and 7 days after the second P addition (*open bars*, *p* = 0.0529). Plants were inoculated with fungi and grown as indicated in the legend to Fig. 1. *Bars of each type labelled with the same letter* are not significantly different at the *p* < 0.05 level. *Letters above the open bars* correspond to the values for incremental P concentration 7 days after the pulse. *Error bars* are SE (*n* = 3)

The shoot P concentration of jarrah plants was determined before and after adding two pulses of P (Fig. 4). Plants inoculated with *Scleroderma* sp. had significantly (*p* < 0.05) higher shoot P concentration than those inoculated with *R. irregularis* 1 day before addition of the first P pulse; however, there was no significant difference (*p* < 0.05) between shoot P concentrations among other treatments (Fig. 4, open bars). All inoculated treatments except *R. irregularis* had slightly (not significant) higher shoot P concentration than NM controls. There were no toxicity symptoms in any of the treatments 1 day after adding the first P pulse. In each case, the shoot tissues had a concentration of less than 1.8 mg P g^−1^ DW and there was no significant difference across treatments (Fig. 4, checkered bars). The incremental increase in shoot P concentration was significantly lower (*p* < 0.05) in plants inoculated with *A. occidentalis* and *Scleroderma* sp. compared to the NM controls (Fig. 5, closed bars).

The severe P toxicity symptoms in plants 7 days after adding the second P pulse (Fig. 3) correlated with shoot P concentrations ranging from 5.5 to 9.5 mg P g^−1^ DW (Fig. 4, closed bars). Thus, the P toxicity symptoms developed at a shoot P concentration somewhere between 1.8 and 5.5 mg P g^−1^ DW. Generally, there was a moderately well fitted but significant positive linear relationship between shoot P concentration and toxicity symptoms (*r* = 0.62, *p* = 0.0064; data not shown) and the shoot P concentrations trended to be lower for 16-week-old plants inoculated with fungi compared to NM plants 7 days after the addition of the second dose of P (Fig. 4, closed bars); however, only *R. irregularis* and dually inoculated plants had significantly lower shoot P concentration compared to NM seedlings (*p* < 0.05). During the week after the second P addition (between second and third harvests), plants inoculated with *R. irregularis* and the dual inoculum had significantly smaller increases in shoot P concentration (*p* < 0.05) than the NM control plants (Fig. 5, open bars).

### Mycorrhizal fungi can enhance jarrah biomass production

**Fig. 6:**
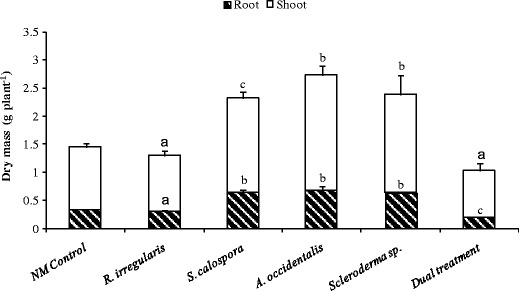
Shoot and root dry mass of jarrah plants after 16 weeks growth and two P fertilizations. Plants were inoculated with fungi and grown as indicated in the legend to Fig. 1. *Letter a* designates values that are not significantly different from the NM control at *p* < 0.10. *Letters b* (*p* = 0.0024 for shoots and *p* = 0.0001 for roots) and **c** (*p* = 0.0109 for shoots and *p* = 0.0001 for roots) represent significant differences from the NM control plants at *p* < 0.05 and *p* < 0.10, respectively. *Error bars* are SE (*n* = 3)

The shoot dry biomass significantly increased in jarrah plants inoculated with *S. calospora* (*p* < 0.10), *A. occidentalis* (*p* < 0.05) or *Scleroderma* sp. (*p* < 0.05) after 16 weeks growth and two P fertilizations, compared to NM controls (Fig. 6). A significant increase was also observed in the root dry biomass of plants inoculated with *S. calospora*, *A. occidentalis* or *Scleroderma* sp., compared to NM plants (*p* < 0.05). However, no positive growth response was observed in *R. irregularis* and dually inoculated treatments. Furthermore, a significant depression of root system growth was observed in seedlings co-inoculated with *R. irregularis* and *Scleroderma* sp. (*p* < 0.10).

## Discussion

### Mycorrhiza and P uptake under P-deficient conditions

The presence of *A. occidentalis* and *Scleroderma* sp. caused a dramatic increase in shoot P content of jarrah plants grown under P-deficient conditions in keeping with the existing literature showing improved plant P nutrition in other eucalypt–ECM symbioses (Bougher et al. 1990; Jones et al. 1998). The facilitation of P uptake by *A. occidentalis* is remarkable as this fungus did not form mycorrhizal structures with jarrah roots; however, fungal hyphae were observed around the roots (Kariman et al. 2012). Furthermore, *A. occidentalis* did not induce short root formation or any other observable morphological changes to the root system. These results indicate that the P uptake of plants does not necessarily correlate with the root colonization ability of fungal partners. Our previous study demonstrated that a higher carboxylate concentration in the rhizosphere soil correlated with the enhanced shoot P content of jarrah plants associated with *A. occidentalis* (Kariman et al. 2014). Here, the improved P nutrition of ECM plants (*Scleroderma* sp.) could be due to exudation of carboxylates to release P from primary minerals (Landeweert et al. 2001) and/or P-mobilizing enzymes that release P from soil organic matter (Bending and Read 1995; Tibbett and Sanders 2002). It would be interesting to investigate how the mentioned mechanisms are regulated under P toxicity conditions.

By 14 weeks, neither AM fungus caused a significant increase in the shoot P content of jarrah plants growing under P-deficient conditions. This result is in agreement with a report showing that AM fungi have a low capacity to improve the P nutrition of eucalypts (Jones et al. 1998). However, at this stage, plants inoculated with *S. calospora* had significantly higher shoot biomass than NM controls, whereas no positive response was observed for the *R. irregularis* treatment (Kariman et al. 2012). In the dual treatment, the AM fungus (*R. irregularis*) seemingly dominated the outcome, as there was neither an increase in the shoot P content nor a positive growth response.

### Mycorrhiza and tolerance to P toxicity

All four fungal isolates tested induced tolerance to P toxicity in jarrah, as judged by the reduction in P toxicity symptoms. The induced tolerance, however, was not always accompanied by a lower shoot P concentration in the inoculated plants. The shoot P concentration of jarrah plants growing under P-deficient conditions was less than 1.5 mg P g^−1^ DW in keeping with previous reports for jarrah (Dell et al. 1987) and *Eucalyptus urophylla* (Aggangan et al. 1996). The symptoms of P toxicity in jarrah had developed at a shoot P concentration between 1.8 and 5.5 mg P g^−1^ DW, indicating that jarrah is a highly P-sensitive species compared to other plants (Shane et al. 2004b and references therein). A single dose of P fertilizer equivalent to an elemental dose of 40 kg ha^−1^ is routinely applied to jarrah forest restoration (Koch and Samsa 2007). While it is impossible to exactly correlate this field dose with our pot growth experiments, especially as the soil conditions differ, it is clear from our results that a very low dose of P can have a harmful effect on jarrah, especially if the plants are not involved in fungal associations. This conclusion then produces an important consideration for the management of jarrah forest restoration.

Smith et al. (2003, 2004) showed that there had been a misevaluation of the contribution made by mycorrhizal fungi to P uptake by host plants, such that the mycorrhiza-mediated P uptake could be much higher than previously presumed. Indeed, P can be almost exclusively supplied to plants via the mycorrhizal pathway for some plant–fungus combinations (Smith et al. 2003, 2004). Accordingly, the low shoot P concentration in plants inoculated with *R. irregularis* (alone or in combination with *Scleroderma* sp.) after the second P pulse could be due to the low contribution of mycorrhizal P uptake pathway, apparently because of very low colonization. Another potential explanation for these results is that colonization caused a down-regulation of P acquisition capacity in jarrah as established for AM symbioses (Liu et al*.* 1998; Burleigh and Harrison 1999; Rosewarne et al*.* 1999; Burleigh 2001; Karandashov and Bucher 2005).

Plants inoculated with *S. calospora*, *A. occidentalis* or *Scleroderma* sp. trended toward a lower shoot P concentration than NM seedlings, apparently resulting from higher biomass. This slight difference in shoot P concentration (20–30 %) may be linked to the reduced toxicity symptoms observed. In keeping with our results, Nazeri et al. (2013) demonstrated that AM symbioses reduced the shoot P concentration after a moderate P pulse (15 mg P kg^−1^ soil) in five legume species including *Kennedia prostrata*, *Cullen australasicum*, *Bituminaria bituminosa*, *Medicago sativa* and *Trifolium subterraneum*, which was due to reduced movement of P from root to shoot. Different combinations of these potential mechanisms of P tolerance might be active to various extents in the different symbioses. Our results indicate that the AM fungus *R. irregularis* is more effective than the tested ECM and non-colonizing fungi at reducing the jarrah shoot P concentration during exposure to elevated P conditions. These results suggest that the AM partner *R. irregularis* may have a low capacity to provide P for the host plant than the ECM and non-colonizing partners.

One implication of our findings is that pre-inoculation of jarrah seedlings in nurseries with both AM and ECM fungi would be helpful in minimizing P toxicity symptoms. This would be applicable where new plantations are being established in soils over-fertilized with P. However, the exotic AM fungus *R. irregularis* had no positive effects on jarrah shoot and root biomass. Furthermore, *R. irregularis* inhibited ECM colonization and function (Kariman et al. 2012) in dually inoculated plants, resulting in the smallest root system amongst treatments, which would affect the establishment and anchoring of plants. For optimal plantation or restoration success, application of mixed endemic AM populations from comparable undisturbed soils should be assessed for effectiveness in terms of both positive growth responses and P tolerance in jarrah.

### Biomass production

Plants inoculated with *A. occidentalis* and *Scleroderma* sp. had the largest biomass among the treatments regardless of their colonization extent confirming the studies showing substantial effects of ECM fungi on eucalypt growth (Bougher et al. 1990; Thomson et al. 1994; Jones et al. 1998). To date, several studies have shown significant contribution of fungi to plant growth by forming non-typical structures or without forming any mycorrhizal structures (Neumann 1959; Warcup and McGee 1983; Kope and Warcup 1986; Kariman et al. 2012, 2014). High concentration of carboxylates in the rhizosphere soil is one of the mechanisms linked with positive growth responses in a symbiosis with no root colonization (Kariman et al. 2014). Protons, phenolic compounds and P-mobilizing enzymes such as acid phosphatases are among the other common factors that enhance P availability in soil leading to improved plant growth and P acquisition (Bending and Read 1995; Landeweert et al. 2001; Tibbett and Sanders 2002).

Two AM fungi had contrasting effects on jarrah growth and the positive growth response was only observed with *S. calospora*, which had relatively higher AM colonization than the other AM treatments. Nevertheless, the extent of root colonization does not necessarily have a direct correlation with positive growth and nutritional responses (Jakobsen 1995; Smith et al. 2004). It seems that the growth response in AM–eucalypt symbioses is strongly dependent on the AM fungus, the plant species and the experimental conditions. The response of eucalypts to AM inoculation has been a matter of controversy during the past decades due to inconsistent results. Gomez et al. (1987) observed no growth stimulation in eight *Eucalyptus* species 3 months after inoculation with 30 AM isolates. Other studies, however, reported positive effects of AM fungi on growth of different *Eucalyptus* species (Adjoud et al. 1996; Chen et al. 2000). This study confirms the inconsistency of growth response in AM–eucalypt symbioses by showing that two different AM isolates have opposite effects on jarrah.

In conclusion, we demonstrated that AM, ECM and the *A. occidentalis* associations could induce tolerance to elevated P in jarrah plants. The protective effect was not always accompanied by a significant reduction in shoot P concentration, and it was independent of the type of fungal association and the extent of root colonization. Moreover, *A. occidentalis* and *Scleroderma* sp. formed more effective symbioses with jarrah plants in terms of plant growth benefits and P nutrition under P-deficient conditions. Finally, the findings suggest that pre-inoculation of jarrah seedlings with symbiotic fungi could be a potential strategy to reduce P toxicity symptoms in plants grown on soils disturbed by over-fertilization.
